# A new method for class prediction based on signed-rank algorithms applied to Affymetrix^® ^microarray experiments

**DOI:** 10.1186/1471-2105-9-16

**Published:** 2008-01-11

**Authors:** Thierry Rème, Dirk Hose, John De Vos, Aurélien Vassal, Pierre-Olivier Poulain, Véronique Pantesco, Hartmut Goldschmidt, Bernard Klein

**Affiliations:** 1 INSERM, U847, 99 rue Puech Villa, 34197 Montpellier, France; 2 CHU-Montpellier, Institute of Research in Biotherapy, Hôpital Saint-Eloi, 34295 Montpellier, France; 3 Medizinische Klinik und Polyklinik V, Universitätsklinikum, Heidelberg, Germany

## Abstract

**Background:**

The huge amount of data generated by DNA chips is a powerful basis to classify various pathologies. However, constant evolution of microarray technology makes it difficult to mix data from different chip types for class prediction of limited sample populations. Affymetrix^® ^technology provides both a quantitative fluorescence signal and a decision (*detection call*: absent or present) based on signed-rank algorithms applied to several hybridization repeats of each gene, with a per-chip normalization. We developed a new prediction method for class belonging based on the detection call only from recent Affymetrix chip type. Biological data were obtained by hybridization on U133A, U133B and U133Plus 2.0 microarrays of purified normal B cells and cells from three independent groups of multiple myeloma (MM) patients.

**Results:**

After a call-based data reduction step to filter out non class-discriminative probe sets, the gene list obtained was reduced to a predictor with correction for multiple testing by iterative deletion of probe sets that sequentially improve inter-class comparisons and their significance. The error rate of the method was determined using leave-one-out and 5-fold cross-validation. It was successfully applied to (i) determine a sex predictor with the normal donor group classifying gender with no error in all patient groups except for male MM samples with a Y chromosome deletion, (ii) predict the immunoglobulin light and heavy chains expressed by the malignant myeloma clones of the validation group and (iii) predict sex, light and heavy chain nature for every new patient. Finally, this method was shown powerful when compared to the popular classification method Prediction Analysis of Microarray (PAM).

**Conclusion:**

This normalization-free method is routinely used for quality control and correction of collection errors in patient reports to clinicians. It can be easily extended to multiple class prediction suitable with clinical groups, and looks particularly promising through international cooperative projects like the "Microarray Quality Control project of US FDA" MAQC as a predictive classifier for diagnostic, prognostic and response to treatment. Finally, it can be used as a powerful tool to mine published data generated on Affymetrix systems and more generally classify samples with binary feature values.

## Background

In allowing simultaneous quantification of the expression level of thousands of genes, DNA chip technology is part of the revolution in molecular biology towards a comprehensive understanding of cell biology at the genome scale, with considerable stake in improving patient classification [1] and treatment. But the huge mass of information from chips has generated a number of difficulties in interpreting results, accentuated by both biological and technical sources of variability [2-5]. However, this technology is the only way to dissect biological pathways [6] and distinguish statistically significant differences in pangenomic gene expression in a single experiment.

Unsupervised analysis provides patient groups that are then compared by supervised analysis, like support vector machines [7], classification trees [8], neural networks [9] or shrunken centroids [10], and leading to functional gene signatures for hematological malignancies [11-16]. Most importantly for clinical practice, the prediction of sample classes occurs whereby a classification system is trained by a known data set, then tested on a validation set, and finally used to predict classification [17-19], prognosis [20-26] or response to treatment [27] for new hematology patients, with careful validation procedures [28].

However, all of the previously published methods for supervised classification and prediction are based on fluorescence signal values, making all results dependent on the way individual chips in an experiment are normalized using one of the numerous low or high-level normalization methods (Global scaling, MAS5, MBEI, RMA, GCRMA, PLIER, [29]). Affymetrix^® ^technology provides both a quantitative fluorescence signal and a decision (present (P) or absent (A) call) based on signed-rank algorithms [30] applied to several spread hybridization repeats of matched and mismatched probes of each gene, with possible regional bias [31]. To skip the inter-chip normalization step [32] and to make the method independent of the chip type, we developed a new prediction method for class belonging based on a statistically-assessed binary criterion of presence/absence of genes instead of expression levels, after normalization with MAS5 or higher. Biological data from normal donors [33] and three groups of newly-diagnosed multiple myeloma (MM) patients considered training and predicted groups, were obtained as previously described [34-36] and statistical issues were addressed by Bonferroni correction for multiple testing, leave-one-out and 5-fold cross-validation and validation with independent data [37]. The present paper reports the development of such predictors on trivial data (sex determination) and a simple clinical application (immunoglobulin light and heavy chain determination). Training is achieved on data from different pooled chip types, and reveals powerful predictive capabilities when compared to the widely used Prediction Analysis of Microarrays (PAM, [38]) run in parallel on the Affymetrix-normalized signals. Important applications potentially derived from this method for high throughput diagnostic, prognostic and drug response determinations point to a-la-carte treatment of cancer based on microarray data obtained at the time of diagnosis.

## Results

### Predictor building

Training data were obtained by pooling samples from hybridizations either on both A and B chips (noted A+B) or P chips, having 44,754 probe sets in common, named "AB+P" set thereafter.

Each class is a collection of sample vectors containing binary variables: 1 for presence or 0 for absence for probe sets from the AB+P list.

A preliminary step to reduce the length of sample vectors and hence computational time is to shorten the initial gene list. This is readily obtained first by filtering out probe sets with no presence in samples, and second by keeping the most class-discriminating probe sets based on a *χ*^2 ^test comparing the occurrences of presence/absence (1/0) among classes.

Every sample of a class is then compared to every sample of the other for the expression of each probe set by creating a "XOR" differential vector (vector values set to 1 if sample calls are different, and 0 if identical). A *χ*^2 ^calculation on the occurrences of 1 is made between the differential vector and the null vector of same length. A sample to sample comparison for a set of genes is therefore characterized by first: a significance decision (non significant = 0, significant = 1) if the *χ*^2 ^is reached for a given, Bonferroni-corrected *P *value (i.e. *P *value/vector length), and second: the *χ*^2 ^value itself corrected for the vector length (named ${Χ}^{2}=\frac{\chi^{2}}{g}$) as an indicator of significance strength. The final class comparison consists of three values, the sum of all individual significance decisions (named NS), the overall strength as the sum of all X^2 ^(named f) and finally the smallest X^2 ^for all the individual comparisons (named X^2^_min_). For a given gene list, those three values are initialized. Deletion of a gene without predictive power from the starting list of genes would result in improving at least one of the three preceding values. The principle of the list reduction to a predictor is therefore to remove each probe set one after the other from the initial list in order to compute the modifications of the three preceding values before returning it to the list, and definitely delete from the list the probe set the removal of which leads to the strongest improvement. The process stops when no further improvement is possible. Mathematical inferences and algoritms are detailed in "Methods".

**Figure 1:**
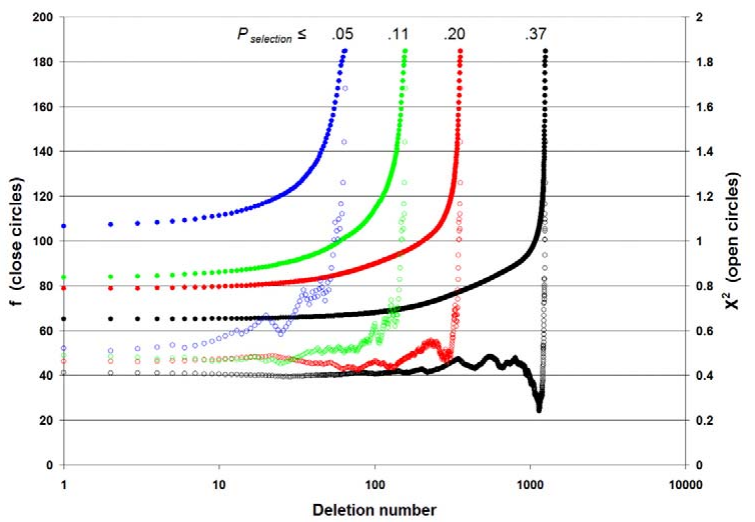
**Effect of stringency of feature dimensionality reduction on predictor construction**. Probe set selection between IgA and IgG heavy chain-expressing MM patient groups over a wide range of *P*_*selection *_values (from .05 to .37, different colors). The number of selected probe sets has no effect on the length and content of the resulting predictor after deletions with a *P*_*build *_value equal to or less than .01 divided by the list length for Bonferroni correction, while the computational time (standard desktop computer) is strikingly reduced. Close circles: f function or overall strength of interclass comparisons on the left vertical scale. Open circles: X^2 ^or $\frac{\chi^{2}}{g}$ min, or smallest strength of all interclass comparisons on the right vertical scale. The number of non-significant interclass comparisons NS is null here.

Whatever the stringency of the *P *value (noted *P*_selection_) for the data reduction step, the final predictor has the same length and content. When there are no longer non-significant comparisons between classes, deletions occur only by increasing f or X^2^_min_. Figure 1 displays the evolution of f and X^2^_min _in the case of training a sex predictor. Selection was made for *P*_selection _values from .05 to .37, leading to initial lists from 77 to 1,267 probe sets. The deletion process performed at a constant *P *value of .01 before Bonferroni correction produced an identical 12 probe set predictor. However, the calculation time has been decreased by more than 3,000 times over the *P*_selection _value range.

### Sex prediction

The present predictor building method was applied to predict sex by training with 21 samples of purified populations of memory B cells, bone marrow plasma cells and polyclonal plasma cells of healthy individuals separated into gender classes of 10 women and 11 men, respectively, and hybridized on A+B chips. With a *P*_selection _value set as described in previous section for discriminating the starting probe set selection, the final predictor found using Bonferroni correction was a short list 12 probe sets encompassing 7 genes, all of which being not surprisingly located on the sex chromosomes. The predictor included the XIST gene, clearly expressed by female samples, as well as genes located on the Y chromosome and expressed by male samples. Five commercial RNA extracted from testis and hybridized in the same conditions were submitted to classification by successive introduction into either gender class. Calculation of the resulting non-significant comparisons (see Methods) resulted in classification as male with no error (data not shown). Leave-one-out cross validation was performed with the 21 possible sample removals and the whole process of establishing a discriminative gene list then deleting from it for predictor building was run, resulting in no classification error when left-out sample was returned to the correct gender class.

This predictor was then applied to the 68 MM patient group hybridized on P chips, by successively introducing them into M or F gender class, and calculating the corresponding NS. Table 1 shows that 67/68 female patients were accurately classified. The unclassified sample was rejected from male patients by Y chromosome absence, but was excluded from women because of a too low XIST gene level on P chips for "present" status. Twenty-seven male patients out of a total of 34 were correctly classified as men, while the remaining 7 were rejected as male by non significant interclass comparisons, six being rejected by both gender classes and the other classified as a woman. In order to check for the male status of these misclassified patients, we used a standard short tandem repeat analysis that clearly evidenced a partial to complete loss of the Y chromosome, as previously observed for about 20% of the elder MM patients [39]. Thus, the present method allows to sort out these male patients with such a loss of Y chromosome.

**Table 1:** Prediction in biological assessment. Summary of the light and heavy chain and sex prediction obtained for 47 new patients.

Patient	Sex							Light chain							Heavy chain						
	NS errors			PAM score			File	NS errors			PAM score			File	NS errors			PAM score			File
	F	M	P	F	M	P		κ	λ	P	κ	λ	P		A	G	P	A	G	P	
E4006	11	0	M	0.000	1.000	M	M	9	3	λ	0.000	1.000	λ	λ	***9***	***1***	***G***	1.000	0.000	A	A
E4020	0	10	F	***0.168***	***0.832***	***M***	F	11	1	λ	0.000	1.000	λ	λ	23	0	G	0.000	1.000	G	G
E4038	0	10	F	1.000	0.000	F	F	0	64	κ	1.000	0.000	κ	κ	Light chain myeloma						
E4049	0	10	F	***0.000***	***1.000***	***M***	F	0	52	κ	1.000	0.000	κ	κ	Light chain myeloma						
E4050	11	10	MY-	0.000	1.000	MY-	MY-	1	50	κ	1.000	0.000	κ	κ	15	0	G	0.000	1.000	G	G
E4054	11	0	M	0.000	1.000	M	M	0	48	κ	0.903	0.097	κ	κ	41*	61*	A	***0.077***	***0.923***	***G***	A
E4055	11	0	M	0.000	1.000	M	M	23	0	λ	0.000	1.000	λ	λ	56	1	G	0.000	1.000	G	G
E4056	0	10	F	1.000	0.000	F	F	0	55	κ	1.000	0.000	κ	κ	1	2	A	1.000	0.000	A	A
E4057	11	0	M	0.000	1.000	M	M	7	3	λ	0.008	0.992	λ	λ	29	0	G	***0.995***	***0.005***	***A***	G
E4060	11	0	M	0.000	1.000	M	M	0	35	κ	1.000	0.000	κ	κ	48	0	G	0.000	1.000	G	G
E4067	11	0	M	0.000	1.000	M	M	0	50	κ	1.000	0.000	κ	κ	57	0	G	0.000	1.000	G	G
E4071	11	0	M	0.000	1.000	M	M	0	51	κ	1.000	0.000	κ	κ	59	0	G	0.000	1.000	G	G
E4073	0	10	MY-	0.000	1.000	MY-	MY-	0	58	κ	1.000	0.000	κ	κ	48	0	G	0.000	1.000	G	G
E4078	0	10	F	***0.000***	***1.000***	***M***	F	8	1	λ	0.000	1.000	λ	λ	IgD myeloma						
E4085	0	10	F	***0.001***	***0.999***	***M***	F	15	2	λ	0.003	0.997	λ	λ	Light chain myeloma						
E4094	11	0	M	0.000	1.000	M	M	0	51	κ	0.770	0.230	κ	κ	0	17	A	***0.254***	***0.746***	***G***	A
E4105	0	10	F	***0.000***	***1.000***	***M***	F	6	3	λ	0.000	1.000	λ	λ	Light chain myeloma						
E4106	0	10	F	0.996	0.004	F	F	0	42	κ	0.997	0.003	κ	κ	0	5	A	***0.065***	***0.935***	***G***	A
E4121	0	10	F	***0.000***	***1.000***	***M***	F	27	0	λ	0.000	1.000	λ	λ	27	0	G	0.000	1.000	G	G
E4122	11	10	MY-	0.000	1.000	MY-	MY-	0	59	κ	0.999	0.001	κ	κ	0	16	A	1.000	0.000	A	A
E4126	11	0	M	0.000	1.000	M	M	0	50	κ	0.983	0.017	κ	κ	0	17	A	1.000	0.000	A	A
E5007	0	10	F	***0.000***	***1.000***	***M***	F	0	32	κ	1.000	0.000	κ	κ	2	3	A	***0.291***	***0.709***	***G***	A
E5024	11	0	M	0.000	1.000	M	M	0	51	κ	0.999	0.001	κ	κ	IgD myeloma						
E5029	0	10	F	***0.000***	***1.000***	***M***	F	28	0	λ	0.000	1.000	λ	λ	Light chain myeloma						
E5035	0	10	F	1.000	0.000	F	F	0	56	κ	0.994	0.006	κ	κ	52	0	G	0.000	1.000	G	G
E5038	11	10	MY-	0.000	1.000	MY-	MY-	0	59	κ	0.974	0.026	κ	κ	Light chain myeloma						
E5040	0	10	F	***0.001***	***0.999***	***M***	F	1	38	κ	0.998	0.002	κ	κ	96*	43*	G	0.000	1.000	G	G
E5043	0	10	F	***0.001***	***0.999***	***M***	F	0	48	κ	0.994	0.006	κ	κ	45	0	G	0.000	1.000	G	G
E5046	0	10	F	***0.000***	***1.000***	***M***	F	28	0	λ	0.000	1.000	λ	λ	0	19	A	1.000	0.000	A	A
E5048	11	0	M	0.000	1.000	M	M	25	0	λ	0.000	1.000	λ	λ	17	0	G	0.000	1.000	G	G
E5049	0	10	F	***0.001***	***0.999***	***M***	F	1	48	κ	0.996	0.004	κ	κ	52	0	G	0.000	1.000	G	G
E5065	11	0	M	0.000	1.000	M	M	25	0	λ	0.001	0.999	λ	λ	Light chain myeloma						
E5066	0	10	F	***0.000***	***1.000***	***M***	F	28	0	λ	0.008	0.992	λ	λ	Light chain myeloma						
E5068	0	10	F	***0.000***	***1.000***	***M***	F	16	0	λ	0.122	0.878	λ	λ	0	17	A	0.875	0.125	A	A
E5069	0	10	F	***0.001***	***0.999***	***M***	F	0	62	κ	0.695	0.305	κ	κ	104*	44*	G	0.000	1.000	G	G
E5081	0	10	F	***0.000***	***1.000***	***M***	F	26	0	λ	0.212	0.788	λ	λ	33	0	G	0.000	1.000	G	G
E5084	11	10	MY-	0.000	1.000	MY-	MY-	23	0	λ	0.088	0.912	λ	λ	0	18	A	***0.019***	***0.981***	***G***	A
E5087	0	10	F	1.000	0.000	F	F	0	61	κ	0.912	0.088	κ	κ	55	0	G	0.000	1.000	G	G
E5093	0	10	F	0.999	0.001	F	F	1	35	κ	0.978	0.022	κ	κ	43	0	G	0.000	1.000	G	G
E5103	0	10	F	1.000	0.000	F	F	1	39	κ	0.997	0.003	κ	κ	47	0	G	0.000	1.000	G	G
E5104	11	0	M	0.000	1.000	M	M	0	51	κ	0.927	0.073	κ	κ	Light chain myeloma						
E5106	0	10	F	***0.002***	***0.998***	***M***	F	0	58	κ	0.873	0.127	κ	κ	0	7	A	0.552	0.448	A	A
E5125	11	0	M	0.000	1.000	M	M	3	13	κ	0.841	0.159	κ	κ	5	1	G	0.000	1.000	G	G
E5126	11	10	MY-	0.000	1.000	MY-	MY-	0	38	κ	***0.236***	***0.764***	***λ ***	κ	9	0	G	0.000	1.000	G	G
E5136	0	10	F	1.000	0.000	F	F	0	59	κ	***0.391***	***0.609***	***λ ***	κ	Light chain myeloma						
E5138	11	0	M	0.000	1.000	M	M	17	1	λ	0.000	1.000	λ	λ	Light chain myeloma						
E5139	0	10	F	***0.003***	***0.997***	***M***	F	11	1	λ	0.000	1.000	λ	λ	21	0	G	0.000	1.000	G	G
E6002	11	0	M	0.000	1.000	M	M	0	46	κ	0.958	0.042	κ	κ	Light chain myeloma						
E6003	11	0	M	0.000	1.000	M	M	0	53	κ	0.975	0.025	κ	κ	44*	65*	A	0.855	0.145	A	A
E6008	11	0	M	0.000	1.000	M	M	0	26	κ	0.891	0.109	κ	κ	29	1	G	0.000	1.000	G	G
E6011	11	0	M	0.000	1.000	M	M	0	56	κ	0.975	0.025	κ	κ	49	0	G	0.000	1.000	G	G
E6020	***11***	***10***	***MY-***	***0.000***	***1.000***	***M***	F	16	0	λ	0.000	1.000	λ	λ	6	0	G	0.000	1.000	G	G
E6022	0	10	F	1.000	0.000	F	F	0	39	κ	1.000	0.000	κ	κ	0	4	A	1.000	0.000	A	A
E6024	11	0	M	0.000	1.000	M	M	10	1	λ	0.000	1.000	λ	λ	IgD myeloma						
E6025	11	0	M	0.000	1.000	M	M	4	13	κ	***0.003***	***0.997***	***λ ***	κ	30	1	G	***1.000***	***0.000***	***A***	G
E6026	0	10	F	***0.001***	***0.999***	***M***	F	0	27	κ	1.000	0.000	κ	κ	36	0	G	0.000	1.000	G	G
E6049	11	0	M	0.000	1.000	M	M	0	12	κ	1.000	0.000	κ	κ	2	4	A	***0.008***	***0.992***	***G***	A
E6054	11	0	M	0.000	1.000	M	M	0	40	κ	1.000	0.000	κ	κ	3	8	A	***0.002***	***0.998***	***G***	A
E6056	11	0	M	0.000	1.000	M	M	0	37	κ	1.000	0.000	κ	κ	0	6	A	1.000	0.000	A	A
E6063	11	10	MY-	0.000	1.000	MY-	MY-	1	13	κ	0.999	0.001	κ	κ	8	1	G	0.000	1.000	G	G
E6074	11	0	M	0.000	1.000	M	M	0	22	κ	1.000	0.000	κ	κ	Light chain myeloma						
E6077	11	0	M	0.000	1.000	M	M	1	31	κ	1.000	0.000	κ	κ	51	0	G	0.000	1.000	G	G
E6087	0	10	F	***0.002***	***0.998***	***M***	F	15	1	λ	0.000	1.000	λ	λ	0	8	A	1.000	0.000	A	A
E6092	0	10	F	1.000	0.000	F	F	17	1	λ	0.000	1.000	λ	λ	67*	50*	G	0.000	1.000	G	G
E6100	0	10	F	1.000	0.000	F	F	1	25	κ	***0.477***	***0.523***	***λ ***	κ	70*	44*	G	0.000	1.000	G	G
E6108	0	10	F	1.000	0.000	F	F	0	46	κ	1.000	0.000	κ	κ	Light chain myeloma						
E6117	0	10	F	***0.009***	***0.991***	***M***	F	13	1	λ	0.001	0.999	λ	λ	Light chain myeloma						
E6120	11	0	M	0.000	1.000	M	M	16	2	λ	0.000	1.000	λ	λ	0	2	A	1.000	0.000	A	A

Abbreviations:

F: female, M: male

P: predicted

MY-: male with Y chromosome deletion

A: IgA, G: IgG

*: When the number of non significant comparisons is identical in both classes for the call predictor at a given precision of the Bonferroni-corrected *χ*^2 ^sample-to-sample comparisons, the *P*-value is increased by one log unit, adding intra-class errors to interclass ones, but still leading to a correct classification.

The signal data from the same patients used for training and testing were then applied to PAM Version 2.1 following the software recommendations. An error threshold of 4.4 was chosen both to minimize individual and overall misclassification errors in cross-validation when training, and to ensure a comparable predictor length. While the same five genes are common to both predictors, the PAM one contains six probe sets for the XIST gene. Applying this predictor to the 68 MM patient test group showed that if all male patients were correctly classified independently of Y chromosome deletion, only 12 women out of 34 were classified as such, while the remaining 22 were classified as men. As preceding, low signals of the XIST gene on P chips, representing here 50% of the predictor probe sets, could explain the Y chromosome overvalue and underline the weakness of using signals through different chip types.

### Monoclonal Ig light chain prediction

When we focused on predicting immunoglobulin chains of monoclonal malignant plasma cell proliferation, training for light chain prediction was achieved with 100 MM patients, expressing 69 kappa (43 A+B chips and 26 P chips) and 31 lambda (20 A+B chips and 11 P chips) monoclonal immunoglobulin light chains as assessed by immunoelectrophoresis. This proportion is in agreement with the usual one third lambda/two third kappa light chain distribution in MM [40]. Using either *P*_selection _≤ 10^-4 ^or 10^-3 ^for *χ*^2 ^analysis of discriminative probe sets on the sample classes led to starting lists of 264 or 442 probe sets. Initial evaluation of interclass comparisons was then performed using a *P *value (noted *P*_build_) ≤ .01 for *χ*^2 ^calculation, corrected for multiple testing by dividing the precision by the length of the probe list. The 2139 sample-to-sample comparisons were all significant with a starting 264 probe set list. So the mechanism by which deletions reduced the list to a final 33 probe set predictor implied 226 deletions by maximizing the fmax function, then 5 deletions by maximizing X^2^. The same predictor was obtained with the 442 probe set list, but the computing time was 5 times longer. Calculation of the error score (NS = 0) clearly showed that lambda light chains could be distinguished from kappa without errors at equal to or less than .01 risk, regardless of disease status, the associated heavy chain, or the presence of Bence-Jones chains. Leave-one-out cross-validation was performed for each lambda and kappa samples through the whole procedure from selection of the discriminative probe set list to probe set deletion from that list, generating 100 predictors, all of which classifying the left-out sample without error when comparing the NS between the correct and the erroneous sample reintroduction. Five-fold cross-validation was performed in the same way by separating patients into five groups and successively testing on each group the predictor trained on the others. Three samples out of 100 were misclassified. Finally, the same sample classes were subjected to a PAM analysis using the Affymetrix MAS5 or GCOS-normalized signals without further modifications. After cross-validation, the error threshold was set to minimize misclassification errors in training and led to a 33 probe sets predictor close to the 33 probe sets predictor obtained by our method. Both predictors were then applied to the 68 MM patient group hybridized on P chips. For the call predictor, each new sample was successively introduced into light chain classes, and the corresponding NS was calculated. Table 1 shows that the call predictor made no error, while 4/68 patients were misclassified by PAM as lambda when kappa.

### Monoclonal Ig heavy chain prediction

Training and validation were achieved under the same conditions as described above for the light chains with a 94 patient training group containing 28 IgA (17 A+B chips and 11 P chips) and 66 IgG (34 A+B chips and 32 P chips) monoclonal immunoglobulin heavy chains as assessed by immunoelectrophoresis, a consistent proportion for MM patients. A 38 probe set predictor was extracted with Bonferroni correction from a starting 225 probe set list, with no non-significant interclass comparison. Leave-one-out cross-validation was performed with 94 sub-predictors, making no classifying error when correctly reintroducing the left-out sample. Data from the test group were processed as previously for call predictor and PAM classification, excluding the light chain and IgD myeloma patients. Table 1 displays one classification error (2%) for the present method versus 9 (18%) for PAM. When the number of non significant comparisons is identical in both classes for the call predictor and hampers the classification decision, the stringency of the Bonferroni-corrected *χ*^2^sample-to-sample comparisons is increased by one log unit. Requirements in sample differences increase, adding intra-class errors to interclass ones, but still leading to a correct classification.

## Discussion

Microarray technology is rapidly evolving. In order to be compared, gene expression profiling experiments should be performed with the same type of chips and normalized with the same method. This hampers the use of gene expression data obtained from different microarrays and studies. The present paper describes a new class predictor based on the Affymetrix call, making it possible to put together data from different Affymetrix microarray types. The call is complementary to the fluorescence signal measured in arbitrary units and indicates that a gene has a certain probability of being present (biologically expressed) in or absent from a sample. The simultaneous hybridization to a series of perfectly matched and mismatched probes allows one to estimate local noise to threshold the expression and make a decision on the presence [30]. Due to the increasing chip density, the number of match-mismatch repeats decreases as the number of probed genes increases and technology improves, but nonetheless the *detection call *strategy is kept by Affymetrix. Therefore, experiments performed on different Affymetrix chip types should be comparable, provide they are normalized with compatible software (MAS5 and GCOS). The availability of data for both training and testing is constantly growing but keeping with ascendant compatibility. In spite of controversial use of negative matches, Affymetrix was the only way to provide a P/A algorithm until recent PAN-P development using negative probe sets. This predictor method could now be applied to other microarray systems since the PAN-P algorithm allows to allocate a P/A call to microarray signal data [41]. Thorough signal normalization [42] is necessary to deal with sample preparation, hybridization, washing and scanning variability. Our technique using the Affymetrix decision call avoids this hampering step, but on the other hand, puts on the same level call-decided present genes with highly variable expression (from 50 to 10,000 arbitrary fluorescence units), leading to the same weighting being given to genes in predictors that have highly dispersed expression. In addition, a gene was considered absent or present only by relying on the MAS5 or GCOS decision. Cut-offs of *P*-values for detection calls were set at Affymetrix default values, with marginal calls considered absent calls, although more recent techniques are now available [43]. A and B chips were used in parallel although highly expressed genes are overexpressed on the A chip compared to the B, which contains many genes that are rarely expressed. However, using the detection call overrides artificial inflating of the B chip intensities relative to the A ones.

Bonferroni correction was applied to account for multiple testing. While conservative, this technique is appropriate for selection from probe set lists large enough to prevent low sensitivity, and allowed us to show that by applying the predictor to a completely independent validation set, the built predictors were highly reliable, with sensitivity and specificity very close to 100%. The presence of outliers in immunoglobulin chain isotype detection was readily detected without a further specialized method [44], and confirmed by reassessment of biological data.

Beyond the initial step of data binarization, which is Affymetrix-specific, selection of and deletion from the probe set list by considering that each sample group is a drawing of presence or absence of a gene list is a solution to the more general problem of classification with limited cardinality (few samples) and high dimensionality (many features, here genes or probe sets), making it possible to extend the present method to any classification of groups containing vectors of binary data. A preliminary process of dimensionality reduction is required [45]. In order to avoid dilution by uninformative genes, a first possibility is to select probe sets on the basis of their class discriminant capability, as measured in the present method by a *χ*^2 ^test on binary values, or on continuous signal values with other statistics [17]. For such a reduction, PAM uses a semi-supervised technique "shrinking" class centroids to the overall centroid for each probe set [10]. On the contrary, to preserve information from all probe sets, a second possibility is to transform the large feature space into a smaller one by a limited number of combinations of individual information, like principal component analysis in the SIMCA method [46].

In order to use a *χ*^2 ^table, the calculated presence content of a class should not be less than 5, otherwise the class should be combined with another one to reach the threshold. In this respect, some of our sample groups approach such a situation. The importance of the selection step is stressed in Results: the number of selected probe sets influences the computational time without affecting the length and quality of the deduced predictor. The deletion and optimization process is in the order of (starting length)^2 ^and the number of comparisons for each deletion increases as the product of each class content, practically restricting this starting length to less than 1,000. Probe sets are then individually removed from the selection list and the resulting significance of inter-class comparisons is evaluated with the remaining list. The initial number of non significant sample to sample comparisons NS is almost always null, since *χ*^2 ^tends to be equal to the number of "1" in differential vectors when their length increases. Therefore NS must be the first process cut-off if increased by any further deletion. If NS is unchanged, the second priority is the overall improvement of the significance, i.e. an increase in the sum f of residues, because it underlines the effect of a deletion on all comparisons simultaneously. And actually, if that priority level is given to improvement in the smallest residue, the final predictor is longer and less performing. Since deletion decision for a probe set during the training sequence arises from updating maximized criteria between its removal and return, the present method resembles the Forward-Backward algorithm in Hidden Markov Models [47].

The prediction step is achieved by inserting the sample to predict for in each class successively and measuring the number of non significant errors generated by the samples of the other class. A well-classified sample should generate a low to null number of non significant comparisons when compared to the samples of the wrong class.

Prediction for gender or Ig light and heavy chain type was used to test for the method, but it is also useful to generate quality control when running chips on a per-patient basis. The prediction method described here is thus routinely used in our hands for the microarray report we generate for each patient with multiple myeloma at the University Hospital of Montpellier. It works well even with patients expressing Bence-Jones chains. This predictor method should help to select defined sets of genes with efficient prediction potential to design dedicated microarrays for multiplex quantitative assays. However, problems in sex determination in the context of myeloma arise from partial deletions of the Y chromosome [39]. The present method excludes most of these patients from both gender class and allows classifying them as an entity. Predicting chain isotype is straightforward, and may be used in everyday clinical practice. This also emphasizes that the present method is ideally suited for two-class classification by a unique score, when establishing a multiclass predictor needs as much scores as the number of classes minus 1.

Finally, preliminary results in predicting less clear-cut classes like MM clinical stages show that, although the number of starting non-significant errors (NS) is not null, the present deletion process is able to reduce it to zero and further shorten the list by the two other criteria to clinically-relevant predictors.

As predictors are composed of "must be present" and "must be absent" probe sets for a sample group, the "present" part of the predictor is at least partly a signature of the group, a "molecular symptom" as recently suggested for stratification of clinical phenotypes [48]. This was obvious for the sex predictor, where all the genes predicting for male gender were on the Y chromosome, and partly verified in the case of monoclonal component chains.

However, genes selected for prediction need not be biologically relevant. As pointed out by the MicroArray Quality Control-II project [49], validation of classifiers should not involve demonstrating that predictors are "validated biomarkers of disease status", and our method answers most of the evaluation criteria set for classifiers in this project.

In the space of probe sets, each sample could be described by a linear model with detection calls as independent variables. Approaches like the ones used for mapping of categorical traits from quantitative loci [50] could then be applied to generate a threshold model, allowing one to classify a sample independently of previous training or validation groups.

Still, the present classifying method, using already processed call evaluation through standardized tools like MAS5 or GCOS, gives consistent results rapidly after the hybridization of patient samples at diagnostic on recent Affymetrix chip type.

## Conclusion

Because of its superseding capabilities, the present call algorithm-based method looks particularly promising for further applications like diagnostic classification of monoclonal gammopathies, prognostic grouping and prediction of response to treatment. More widely, it can be used as a powerful tool to mine self-generated or literature data on all cancer types. and specially to perform classification of binary feature-containing samples.

## Methods

### Samples and database implementation

The process described herein has been tested on sex, monoclonal light chain and heavy chain prediction. Methods for recruiting patient groups, as well as cDNA preparation and chip hybridization were described elsewhere [34-36]. Quality controls for hybridization were done and passed as recommended by Affymetrix so that poorly hybridized chips containing an excessive number of absent calls were eliminated. Chip scans were saved into text files through MAS5 then GCOS Affymetrix^® ^data treatment and transferred to our RAGE [51] database. All input/output operations and calculations were managed through a web interface by Perl-CGI scripts running on an Apache/Linux server.

### Notations

The probe set list *PS*^*T *^= (*ps*_1_...*ps*_*k*_...*ps*_*g*_) has an initial length *g*_*init *_of 44,928 probes for A+B chips, 54,613 probes for P chips and 44,754 for both A+B and P combined chips. Classes *X*^*T *^= (*x*_1_...*x*_*i*_...*x*_*m*_) and *Y*^*T *^= (*y*_1_...*y*_*i*_...*y*_*n*_) contain samples $x_{i}^{T}=\left(x_{i}d_{1}...x_{i}d_{k}...x_{i}d_{g}\right)$ and $y_{j}^{T}=\left(y_{j}d_{1}...y_{j}d_{k}...y_{j}d_{g}\right)$. *P*-values are noted *P*_*indice*_.

### Step 1 Prediction Process – Filtering class-discriminating probe sets

In order to work on significantly expressed genes only, we decided to keep a two-level presence status, "Present" as 1 and "Else" as 0. So we used cut-off *P*-values for detection calls at more than .04 for both absent and marginal calls, since the default Affymetrix values are between .04 and .06 for marginal and more than .06 for absent. As recommended by others [52], probe sets were filtered by selecting at least one present call across all samples, to avoid working on always-absent genes, as described in Algorithm 1, Appendix.

The number of probe sets decreased from 44,928 to 33,360 for U133A+B chips with one forced presence (default). The decrease rate of gene number was much lower when further increasing the minimal number of present calls. Subsequent filtering was achieved by applying a *χ*^2 ^test to each probe set distribution in sample groups considered as multiple drawings of a two-stage criterion (presence = 1, else = 0), with a user-defined *P*_*selection *_value, as summarized in the Affymetrix-independent algorithm developed in Algorithm 2, Appendix.

With a user-defined cut-off for *P*_*selection*_, the resulting sorted probe set list is subsequently used for supervised analysis. The *P*_selection _value should be at least .05 to select discriminating probe sets between classes. Decreasing this value results in decreasing the number of selected probe sets by increasing precision. Actually, when many genes are highly differentially expressed between classes, the number of selected probe sets is over 500 at the maximal significance threshold, leading to huge computational time without change in predictive probe sets. Decreasing *P*_selection _value yields to a decrease in number of selected probe sets and deletions, but the final predictor length is constant over a large range of *P*_selection _down to less than or equal to .001 (default value), while the computer time is strikingly decreased for identical probe set content. However, further decrease in *P*_selection _will make the learning process impossible because of a too limited discriminating probe set list with a high rate of non significant interclass comparisons.

### Step 2 Prediction Process – Initializing the discriminating probe set list strength

The principle in evaluating the capacity of a probe set list to separate sample classes is to maximize the significance of sample to sample comparisons using a *χ*^2 ^test. Since detection calls from the same probe set are paired in compared samples, we compare every sample *x*_*i *_of the class *X *to every sample *y*_*j *_of the class *Y *by creating a differential vector **Δ**_ij _whose values are 0 if the two sample detection calls for a probe set are identical, and 1 if they are different. This new vector **Δ**_ij _is then compared to the null vector, representing the H_0 _hypothesis using a *χ*^2 ^test, with a user-defined *P*_build _value with Bonferroni correction for multiple testing and Yates correction for small sample numbers in two class comparisons.

In the **Δ**_ij _vector of *g *elements, the observed number of "1" is *d*_*ij *_and the number of "0" *g *- *d*_*ij*_. The null vector contains *g *"0" only. In both vectors together, containing 2*g *elements, the total number of "1" is *d*_*ij*_, and the total number of "0" 2*g *- *d*_*ij*_, giving the calculated numbers of "1" and "0" in both vectors . The *χ*^2 ^calculation for the ij comparison is straightforward:

(1) $$\begin{array}{c} \chi_{ijYates}^{2}=\frac{{\left(\left|g-d_{ij}-\left(g-\frac{d_{ij}}{2}\right)\right|-\frac{1}{2}\right)}^{2}}{g-\frac{d_{ij}}{2}}+\frac{{\left(\left|g-\left(g-\frac{d_{ij}}{2}\right)\right|-\frac{1}{2}\right)}^{2}}{g-\frac{d_{ij}}{2}}+\frac{{\left(\left|d_{ij}-\frac{d_{ij}}{2}\right|-\frac{1}{2}\right)}^{2}}{\frac{d_{ij}}{2}}+\frac{{\left(\left|-\frac{d_{ij}}{2}\right|-\frac{1}{2}\right)}^{2}}{\frac{d_{ij}}{2}}\\ \chi_{ijYates}^{2}=\frac{2g{\left(d_{ij}-1\right)}^{2}}{d_{ij}\left(2g-d_{ij}\right)} \end{array}$$

If the significance threshold is reached, the samples are not in the same class. This is repeated for comparison of each class sample paired to any sample of the other class and the number of non-significant comparisons NS can be determined. The calculated *χ*^2 ^value is dependent of g, the length of the probe set list. Let's consider instead the expression:

(2) $${Χ}^{2}=\frac{\chi_{ij}^{2}}{g}=\frac{2\frac{d_{ij}}{g}}{2-\frac{d_{ij}}{g}}=2\frac{p_{ij}}{2-p_{ij}}$$

where *p*_*ij *_is the probability of "1" in the differential vector **Δ**_ij_. It is now independent of the number of probe sets in a given list. Every resulting X^2 ^will represent the strength of the sample-to-sample difference. The smallest one, representing the worst of all comparisons, is noted X^2^_min _and the sum of the overall residues for class comparison is represented by the function:

(3) $$f=\sum_{i=1}^{i=m}\sum_{j=1}^{j=n}\frac{\chi_{ij}^{2}}{g}$$

When used for the first time (evaluating the discriminative probe set list), *NS*_0_, *f*_0_, ${Χ}_{0}^{2}$ should be substituted to *NS*, *f*, X^2 ^and *g*_*select *_to *g*_*list *_in Algorithm 3, Appendix.

### Step 3 Prediction Process – Shortening the probe set list by the best deletion

The principle is to minimize the number of non-significant comparisons by successive deletions of the probe set giving the best improvement from the probe set list. For the predictor learning step, a maximum for *P*_build _should also be .05. But slightly scaling down *P*_build _should result in avoiding misclassifications at the validation step when classes present close levels of differential gene expression (e.g. immunoglobulin light-chain cross-validation, *P*_build _≤ .01, default value). However, a strong decrease should delete too few probe sets to make the deletion process valuable.

The step diagram is described in Algorithm 4, Appendix, and the process stops when no criterion can be further improved by probe set removal. The remaining list becomes the predictor.

### Cross-validation

For leave-one-out validation, each sample in turn is removed from its class, and the whole process of dimensionality reduction and predictor building is run with Bonferroni correction on the remaining samples as described for initial classes. Each predictor build in this way is tested for its capacity to generate misclassification errors, *i.e*. the greatest difference in NS when the removed sample is returned either to the class in which it belongs (NS should be 0 or small) or to the other class (NS should be high and ideally equal to the number of samples in the class of origin minus 1).

Five-fold cross validation is done in the same way by dividing the sample population into five groups and testing one in turn with a predictor trained with the four pooled others through the whole process of data reduction and predictor building.

### Prediction

This is achieved in the same way as validation. The new sample is successively added to one of the known classes, and the predictor list is run on both situations (class 1 plus new sample versus class 2, then class 1 versus class 2 plus new sample). The preceding method is run, namely calculating the number of errors generated in both cases by the algorithm #3, the smallest error number assigning the correct classification.

## Authors' contributions

TR conceived the study, created the analysis tools, and drafted the manuscript. DH and HG collected bone marrow samples and clinical data. JDV, AV and POP participated in implementing the analysis tools. VP contributed in performing the chip experiments. BK participated in the research design, analysis and writing. All authors read and approved the final manuscript.

## Appendix

### Algorithm 1. Filtering on presence and data binarization (Affymetrix-specific)

1 **begin initialize**, *k *← 0, *i *← 0, *j *← 0, *g*_*pfilter*_← *g*_*init*_

2   **for ***k *← *k *+1

3      *s*_*k*_← 0

3      **for ***i *← *i *+ 1

4         **if ***ps*_*k *_present in *x*_*i *_**then ***x*_*i *_*d*_*k*_← 1

5         **else ***x*_*i *_*d*_*k*_← 0

6         *s*_*k*_← *s*_*k *_+ *x*_*i *_*d*_*k*_

7      **until ***i *= *m*

8      **for ***j *← *j *+ 1

9         **if ***ps*_*k *_present in *y*_*j *_**then ***y*_*j *_*d*_*k*_← 1

10         **else ***y*_*j *_*d*_*k*_← 0

11         *s*_*k*_← *s*_*k *_+ *y*_*j *_*d*_*k*_

12      **until ***j *= *n*

14      **if ***s*_*k *_= 0 **then **delete *ps*_*k *_from *PS*; *g*_*pfilter*_← *g*_*pfilter *_- 1

13   **until ***k *= *g*_*init*_

16   **return ***PS*, *g*_*pfilter*_

17 **end**

### Algorithm 2. Dimensionality reduction: selecting features (probe sets) discriminating class X from class Y

1 **begin initialize ***P*_*selection*_, *k *← 0, *i *← 0, *j *← 0, *g*_*select*_← *g*_*pfilter*_

2   **for ***k *← *k *+ 1

3      **for ***i *← *i *+ 1

4         *Xo*_*k*_← *Xo*_*k *_+ *x*_*j *_*d*_*k *_(observed)

5      **until ***i *= *m*

6      **for ***j *← *j *+ 1

7         *Yo*_*k*_← *Yo*_*k *_+ *y*_*j *_*d*_*k *_(observed)

8      **until ***j *= *n*

9      $Xc_{k}\leftarrow \frac{m(Xo_{k}+Yo_{k})}{m+n}$ (calculated)

10      $Yc_{k}\leftarrow \frac{n(Xo_{k}+Yo_{k})}{m+n}$ (calculated)

11      Yates-corrected $\chi_{k}^{2}\leftarrow \frac{{\left(Xo_{k}-Xc_{k}-1/2\right)}^{2}}{Xc_{k}}+\frac{{\left(Yo_{k}-Yc_{k}-1/2\right)}^{2}}{Yc_{k}}$

12      **if ***P *($\chi_{k}^{2}$) > *P*_*selection*_

13         **then **delete *ps*_*k *_from *PS*; *g*_*select*_← *g*_*select *_- 1

14   **until ***k *= *g*_*pfilter*_

15   **return ***PS*, *g*_*select*_

16 **end**

### Algorithm 3. Evaluating inter-class comparison for a probe set list of length *g*_*list*_

1 **begin initialize ***P*_*build*_, *NS *← 0, *f *← 0, X^2 ^← 100, *k *← 0, *i *← 0, *j *← 0

2   **for ***i *← *i *+ 1

3      **for ***j *← *j *+ 1

4         *δ *_*ij*_← 0

5         **for ***k *← *k *+ 1

6            **if **(*x*_*i *_*d*_*k*_≠ *y*_*j *_*d*_*k*_) **then ***δ *_*ij*_← *δ *_*ij *_+ 1

7         **until ***k *= *g*_*list*_

8         $\chi_{ij-Yates}^{2}\leftarrow \frac{2g_{list}{\left(\delta_{ij}-1\right)}^{2}}{\delta_{ij}(2g_{list}-\delta_{ij})}$   (Eq. 1)

9         **if **$P(\chi_{ij}^{2})=\frac{P_{build}}{g_{list}}$ (Bonferroni correction)

            **then ***NS *← *NS *+ 1

10         $f\leftarrow f+\frac{\chi_{ij}^{2}}{g_{list}}$   (Eq. 3)

11         **if **$\frac{\chi_{ij}^{2}}{g_{list}}<{Χ}^{2}$**then **${Χ}^{2}\leftarrow \frac{\chi_{ij}^{2}}{g_{list}}$   (Eq. 2)

12      **until ***j *= *n*

13   **until ***i *= *m*

14   **return ***NS*, *f*, X^2^

15 **end**

### Algorithm 4. Reducing the discriminative list to a predictor

1 **begin initialize ***g*_*pred*_← *g*_*select*_, *NS*_min _← *NS*_0_, *f*_max_← *f*_0_, ${Χ}_{\min}^{2}\leftarrow {Χ}_{0}^{2}$

2   **do**

3      *l *← 0

4      *flag *← - 1

5      **for ***l *← *l *+ 1

6         remove *ps*_*l *_from *PS*

7         run algorithm 3 with *g*_*list*_← *g*_*pred*_

8         **if ***NS *<*NS*_min_

            **then ***NS*_min _← *NS*; *f*_max_← *f*; ${Χ}_{\min}^{2}$ ← X^2^; *ps*_*ns*_← *ps*_*l*_; *flag *← 1

9         **elsif ***NS *= *NS*_min _and *f*_max_≤ *f*

            **then ***f*_max_← *f*; ${Χ}_{\min}^{2}$ ← X^2^; *ps*_*f*_← *ps*_*l*_;

               **if ***flag *≠ 1 **then ***flag *← 2

10         **elsif ***NS *= *NS*_min _and *f*_max _> *f *and ${Χ}_{\min}^{2}$ ≥ X^2^

            **then **${Χ}_{\min}^{2}$ ← *ps*_*l*_;

               **if ***flag *≠ 1 and *flag *≠ 2 **then **${Χ}_{\min}^{2}$ ← X^2^; *flag *← 3

11         return *ps*_*l *_to *PS*

12      **until ***l *= *g*_*pred*_

13      **if ***flag *= 1 **then **delete *ps*_*ns *_from *PS*; *g*_*pred*_← *g*_*pred *_- 1

14      **elsif ***flag *= 2 **then **delete *ps*_*f *_from *PS*; *g*_*pred*_← *g*_*pred *_- 1

15      **elsif ***flag *= 3 **then **delete $ps_{{Χ}^{2}}$ from *PS*; *g*_*pred*_← *g*_*pred *_- 1

16   **until ***flag *= - 1

17   **return ***PS *(the final predictor)

18 **end**
